# Effect of calcium phosphate and vitamin D_3_ supplementation on bone remodelling and metabolism of calcium, phosphorus, magnesium and iron

**DOI:** 10.1186/1475-2891-13-6

**Published:** 2014-01-17

**Authors:** Ulrike Trautvetter, Nadja Neef, Matthias Leiterer, Michael Kiehntopf, Jürgen Kratzsch, Gerhard Jahreis

**Affiliations:** 1 Department of Nutritional Physiology, Institute of Nutrition, Friedrich Schiller University of Jena, Dornburger Str, 24, D-07743, Jena, Germany; 2 Thuringian State Institute of Agriculture, Naumburger Str. 98, D-07743, Jena, Germany; 3 Institute of Clinical Chemistry and Laboratory Medicine, Friedrich Schiller University Jena, Erlanger Allee 101, D-07747, Jena, Germany; 4 Institute of Laboratory Medicine, Clinical Chemistry and Molecular Diagnostic, University Leipzig, Liebigstr. 27, D-04103, Leipzig, Germany

**Keywords:** Vitamin D, Calcium, Phosphorus, Magnesium, Iron, Human study, Bone remodelling

## Abstract

**Background:**

The aim of the present study was to determine the effect of calcium phosphate and/or vitamin D_3_ on bone and mineral metabolism.

**Methods:**

Sixty omnivorous healthy subjects participated in the double-blind, placebo-controlled parallel designed study. Supplements were tricalcium phosphate (CaP) and cholecalciferol (vitamin D_3_). At the beginning of the study (baseline), all subjects documented their normal nutritional habits in a dietary record for three successive days. After baseline, subjects were allocated to three intervention groups: CaP (additional 1 g calcium/d), vitamin D_3_ (additional 10 μg/d) and CaP + vitamin D_3_. In the first two weeks, all groups consumed placebo bread, and afterwards, for eight weeks, the test bread according to the intervention group. In the last week of each study period (baseline, placebo, after four and eight weeks of intervention), a faecal (three days) and a urine (24 h) collection and a fasting blood sampling took place. Calcium, phosphorus, magnesium and iron were determined in faeces, urine and blood. Bone formation and resorption markers were analysed in blood and urine.

**Results:**

After four and eight weeks, CaP and CaP + vitamin D_3_ supplementations increased faecal excretion of calcium and phosphorus significantly compared to placebo. Due to the vitamin D_3_ supplementations (vitamin D_3_, CaP + vitamin D_3_), the plasma 25-(OH)D concentration significantly increased after eight weeks compared to placebo. The additional application of CaP led to a significant increase of the 25-(OH)D concentration already after four weeks. Bone resorption and bone formation markers were not influenced by any intervention.

**Conclusions:**

Supplementation with daily 10 μg vitamin D_3_ significantly increases plasma 25-(OH)D concentration. The combination with daily 1 g calcium (as CaP) has a further increasing effect on the 25-(OH)D concentration. Both CaP alone and in combination with vitamin D_3_ have no beneficial effect on bone remodelling markers and on the metabolism of calcium, phosphorus, magnesium and iron.

**Trial registration:**

NCT01297023

## Background

Vitamin D is of large importance for homeostasis of calcium and bone metabolism. For humans, there are two ways for an adequate supply with vitamin D: Firstly, via cutaneous synthesis under UVB exposure, and secondly, via intake with the diet [1]. International scientific literature indicated a worldwide problem of vitamin D deficiency [2]. According to Hintzpeter *et al*., 57% of men and 58% of women in Germany had 25-hydroxyvitamin D (25-(OH)D) concentrations below the recommended concentration of 50 nmol/l [3]. Furthermore, the optimal concentration is defined as a 25-(OH)D > 75 nmol/l [4]. In the first place, vitamin D is responsible for maintaining the extracellular calcium concentrations by controlling absorption of calcium and by direct effects on bone and on parathormone (PTH) secretion [2]. In recent years, vitamin D gained increasing attention because of its association with the risk of overall mortality, diabetes, cancer, musculoskeletal disorders, mental and physical performance, hypertension and cardiovascular diseases [5,6].

The beneficial effects of calcium phosphate mainly focus on the intestinal metabolism, e.g., bile acid metabolism, fatty acid excretion, and modulation of the gut microbiota [7-10]. Calcium from tricalcium phosphate (CaP, a water-insoluble compound at neutral pH value), is partly absorbed in the human gut; but the main part of the calcium and phosphorus is precipitated to amorphous calcium phosphate in the gut, and thus, not absorbed [11]. Nevertheless, CaP showed in combination with vitamin D_3_ beneficial modulation on bone metabolism in elderly women [12]. The aim of the present study was to determine the impact of daily 1 g calcium (as CaP) with or without 10 μg vitamin D_3_ on bone formation and resorption markers as well as on calcium, phosphorus, magnesium and iron metabolism in healthy middle-aged men and women.

## Methods

### Supplements

Two supplements were used in this study: CaP (Ca_5_(PO_4_)_3_OH; cfb, Budenheim, Germany) and cholecalciferol (vitamin D_3_; Brenntag, Mülheim an der Ruhr, Germany). In order to achieve a calcium supplementation of additional 1 g/d and a vitamin D_3_ supplementation of 10 μg/d, CaP and/or vitamin D_3_ were incorporated in wholewheat bread. Participants consumed approximately 135 g of this bread daily. Placebo bread was prepared in the same manner as the test bread, but without CaP and vitamin D_3_. Taste and visual properties of placebo and test breads were comparable.

### Subjects and study design

The study was conducted at the Friedrich Schiller University Jena, Department of Nutritional Physiology, between January and April 2011.

**Figure 1:**
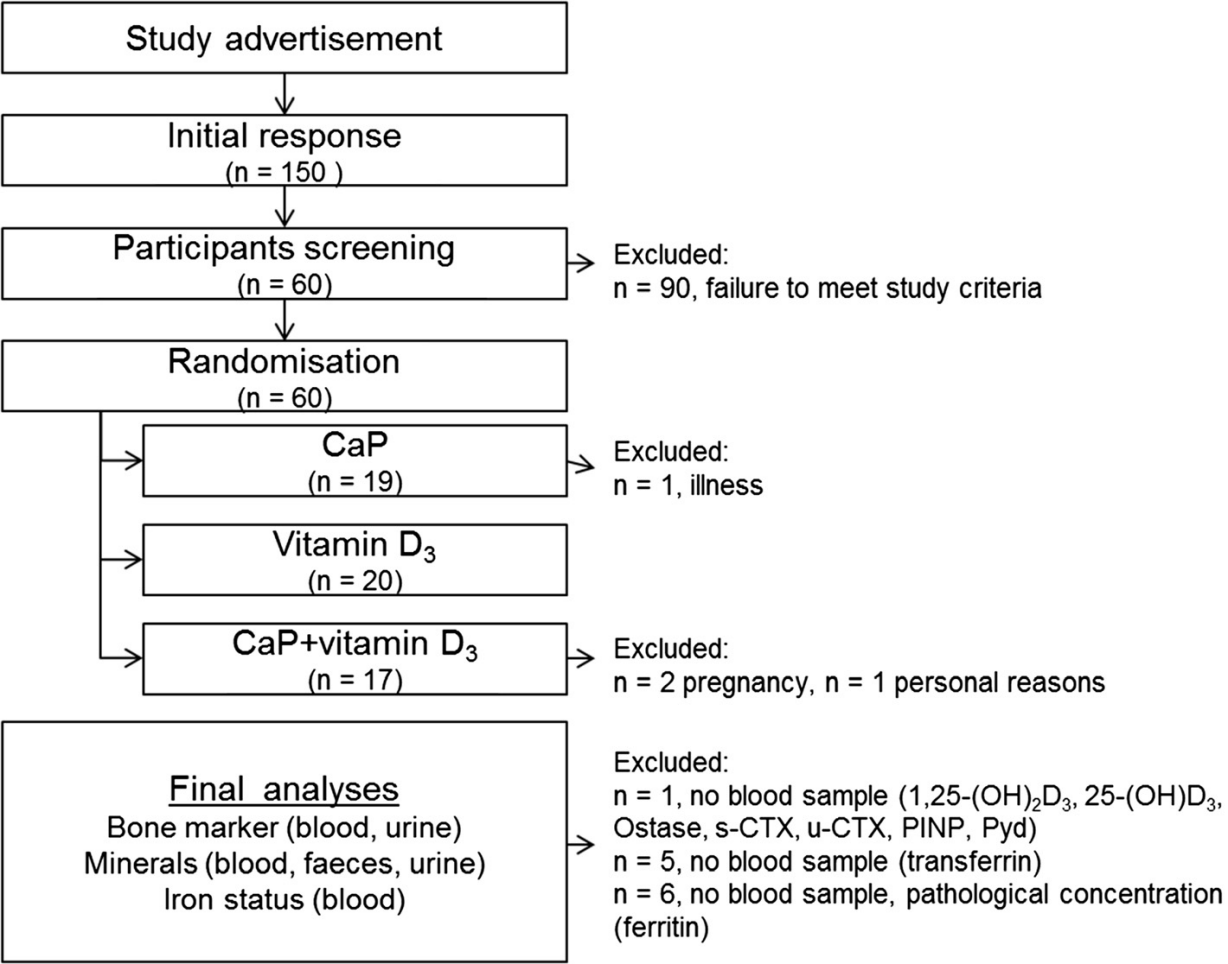
**Flow chart of the study.** CaP: tricalcium phosphate; 1,25-(OH)_2_D: 1alpha,25-dihydroxyvitamin D; 25-(OH)D: 25-hydroxyvitamin D; PTH: parathormone; CTX: cross-linked C-terminal telopeptide of type I collagen; P1NP: N-terminal propeptide of type I procollagen; DPD: desoxypyridinoline.

Sixty omnivorous healthy subjects (men, n = 24; women n = 36) participated in this double-blind, placebo-controlled parallel designed study. Eligibility criteria for participants were age between 20 and 70 years and physical health. All volunteers were informed of the purpose, the course and the possible risks of the study and gave their written consent. The study protocol was approved by the Ethical Committee of the Friedrich Schiller University Jena (No.: 2959-11/10). Four participants dropped out because of pregnancy, illness and personal reasons (Figure 1). The remaining 56 volunteers aged 42 ± 12 y had a BMI of 25 ± 4 kg/m^2^.

At the beginning of the study (baseline), all subjects documented their normal nutritional habits in a dietary record for three successive days. The participants were encouraged to weigh all eaten foods with proved scales. One day later, the subjects collected quantitatively their faeces for three days. From day two to day three of the faeces collection, the subjects collected their urine for 24 h, too. At the last day of the faeces collection, a fasting blood sampling took place. This procedure aimed to familiarise the subjects to the sample collection and to establish a baseline profile of each subject. Afterwards, subjects were allocated to the three intervention groups, so that there were no significant changes in age, BMI and 25-(OH) D concentration between the groups.

After allocation, subjects consumed for two weeks the placebo bread. Then they consumed for eight weeks either the CaP, vitamin D_3_ or the CaP + vitamin D_3_ bread. In the last week of the placebo and after four and eight weeks of intervention, subjects consumed a defined diet for three days. This diet contained all the foods required per subject over the three days and was prepared and pre-weighed in the study centre. The subjects were instructed to consume no other foods than provided. Food intake was calculated by weighing food residues. Samples from each food component were frozen and stored at −20°C until analysis. Furthermore, subjects collected faeces quantitatively for three days, beginning at the second day of the defined diet. From day two to three of the defined diet, the subjects collected their urine for 24 h. On the third day of the defined diet, a fasting venous blood sample was taken.

### Sample preparation

Blood samples were drawn by venipuncture and collected in lithium heparin and serum tubes. Lithium heparin tubes were centrifuged (2500 × *g*, 15 min, 20°C) and the plasma supernatants were stored at −20°C until analysis. Serum tubes were centrifuged (2500 × *g*, 10 min, 20°C) and the serum was stored at −20°C until analysis.

The faecal samples were transported to the study centre without delay. Each specimen was weighed, frozen and stored at −20°C. At the end of the study, faeces samples were homogenised, portioned, and the pH-value was determined. The complete 24 h urine was transported to the study centre at the day of the blood sampling. The urine volume from every participant was measured, and aliquots were frozen at −20°C until analysis.

### Food analysis

The intake of calcium, phosphorus, magnesium, and iron was analysed via ICP-OES as described previously [10]. The intake of energy, fat, proteins, carbohydrates and vitamin D was verified using the Prodi® 5.4 software (Nutri-Science GmbH, Freiburg, Germany). Retained samples of placebo and test breads were homogenised, freeze-dried and analysed for vitamin D_3_ concentration by certified methods (GC-MS) according to the Food GmbH Jena, Analytic and Consulting.

### Blood analysis

Concentrations of calcium, phosphate, magnesium, iron, ferritin and transferrin in plasma and osteocalcin, alkaline phosphatase, 1alpha,25-dihydroxyvitamin D (1,25-(OH)_2_D), 25-(OH)D, PTH and calcitonin in serum were ascertained according to certified methods of the Institute of Clinical Chemistry and Laboratory Medicine, Jena University Hospital.

Serum cross-linked C-terminal telopeptide of type I collagen (CTX) and N-terminal propeptide of type I procollagen (P1NP) were determined using methods according to the Institute of Laboratory Medicine, Clinical Chemistry and Molecular Diagnostic, University Leipzig.

### Faeces and urine analyses

Mineral concentrations in faeces were analysed via ICP-OES as described previously [10].

The concentrations of calcium, phosphorus, magnesium and iron in urine were measured according to certified methods of the Institute of Clinical Chemistry and Laboratory Medicine, Jena University Hospital.

The concentrations of CTX and desoxypyridinoline (DPD) were determined using methods according to the Institute of Laboratory Medicine, Clinical Chemistry and Molecular Diagnostic, University Leipzig.

### Statistics

Samples from each participant were coded to protect volunteer identity and to mask treatment groups during the analysis. All values in the text and tables are means with standard deviations. Data analysis was performed using the statistical software package IBM SPSS Statistics 19 (SPSS Inc. IBM Company, Chicago, USA). Variance homogeneity was tested using the Levene test. In case of variance heterogeneity data were log_10_ transformed (signed with ^t^ in text, tables and figures). The effect of time in each intervention group was tested using general linear model with repeated measurements (with pairwise comparisons based on Fishers-LSD test). The effect of supplementation between groups was tested using univariate analysis of variance followed by Student-Newman-Keuls *post hoc* test. Differences were considered significant at p ≤ 0.05.

## Results

The baseline characteristics of participants of the three intervention groups are presented in Table 1. Due to randomisation, there were no significant differences in age, BMI and 25-(OH)D between intervention groups.

**Table 1:** Baseline characteristics of participants who completed the study

	**CaP**	**Vitamin D**_**3**_	**CaP**+**vitamin D**_**3**_
N	19	20	17
Age [y]	42 (12)	45 (13)	41 (12)
BMI [kg/m^2^]	24 (3)	25 (5)	25 (4)
25-(OH)D [nmol/l]	59 (30)	46 (20)	50 (16)

n = 56; data are expressed as mean (standard deviation); CaP: tricalcium phosphate; 25-(OH)D: 25-hydroxyvitamin D.

### Nutrient intake

There were no differences in the intake of energy, fat, protein, and carbohydrates between study periods (placebo, four and eight weeks) and between intervention groups. The mean intakes of energy, fat, protein, and carbohydrates were 8.7 ± 1.8 MJ/d, 78.6 ± 22.5 g/d, 73.6 ± 12.5 g/d, and 260.2 ± 47.4 g/d, respectively. Due to the CaP and vitamin D_3_ supplementations, the intake of calcium, phosphorus and vitamin D_3_ increased significantly (Table 2). The intake of magnesium and iron did not change between the three study periods and between the interventions. The mean magnesium and iron intakes were 277.8 ± 42.2 mg/d and 7.8 ± 1.1 mg/d, respectively.

**Table 2:** **Mean intake of calcium**, **phosphorus**, **and vitamin D during defined diet in all study periods**

**Parameter**	**Supplements**	**Placebo**	**Interventions**	
			**4 weeks**	**8 weeks**
Calcium [mg/d]	CaP	938^b^ (197)	1998^a^ (192)^1^	2014^a^ (194)^1^
	Vitamin D_3_	905 (187)	897 (219)^2^	916 (203)^2^
	CaP+vitamin D_3_^t^	889^b^ (154)	1913^a^ (151)^1^	1872^a^ (164)^1^
Phosphorous [mg/d]	CaP	1337^b^ (250)	1803^a^ (247)^1^	1833^a^ (242)^1^
	Vitamin D_3_	1311 (236)	1303 (247)^2^	1324 (226)^2^
	CaP+vitamin D_3_	1286^b^ (172)	1733^a^ (180)^1^	1705^a^ (187)^2^
Vitamin D_3_ [μg/d]	CaP	6.5 (0.6)	6.6 (0.6)^1^	6.6 (0.5)^1^
	Vitamin D_3_	6.2^b^ (1.3)	15.2^a^ (1.0)^2^	15.2^a^ (1.0)^2^
	CaP+vitamin D_3_	6.5^b^ (0.4)	15.1^a^ (0.4)^2^	15.1^a^ (0.3)^2^

n = 56; data are expressed as mean (standard deviation); CaP: tricalcium phosphate; ^a b^ mean values within a row with dissimilar superscript letters are significantly different (p ≤ 0.05); ^1 2^ mean values within a column with dissimilar superscript numbers are significantly different (p ≤ 0.05); results without superscripts in a row/column have no significant differences; effect of time was tested using general linear model with repeated measurements (with pairwise comparisons based on Fishers-LSD test); effect of supplementation was tested using univariate analysis of variance followed by Student-Newman-Keuls *post hoc* test.; ^t^ for statistical analysis the data were log_10_ transformed, because of variance heterogeneity.

### Minerals

The plasma calcium concentration decreased significantly in all intervention groups after four and eight weeks of intervention compared to placebo (Table 3; p ≤ 0.05). In all interventions, no effect of time on the renal calcium excretion was observed. After four weeks of intervention, the CaP group had a significantly higher renal calcium excretion compared to vitamin D_3_ (p ≤ 0.05). Due to CaP supplementations (CaP, CaP + vitamin D_3_), faecal excretion of calcium increased significantly after four and eight weeks compared to placebo. The faecal calcium excretion was significantly lower in the vitamin D_3_ group compared to the CaP and CaP + vitamin D_3_ groups at four and eight weeks of intervention (p ≤ 0.05).

**Table 3:** **Concentrations of plasma and excretion of renal and faecal minerals after intervention with either tricalcium phosphate**, **vitamin D**_**3 **_**or both**

**Parameter**	**Supplements**	**Placebo**	**Interventions**	
			**4 weeks**	**8 weeks**
Plasma calcium [mmol/l]	CaP	2.4^a^ (0.1)	2.3^b^ (0.1)	2.3^b^ (0.1)
	Vitamin D_3_	2.4^a^ (0.1)	2.3^b^ (0.1)	2.3^b^ (0.1)
	CaP+vitamin D_3_	2.4^a^ (0.1)	2.3^b^ (0.1)	2.4^b^ (0.1)
Renal calcium excretion [mg/d]	CaP	158 (84)	187 (98)^1^	171 (79)
	Vitamin D_3_	133 (53)	141 (64)^2^	152 (58)
	CaP+vitamin D_3_	117 (60)	143 (63)^12^	130 (57)
Faecal calcium excretion [mg/d]	CaP	763^a^ (434)	1214^b^ (386)^1^	1324^b^ (465)^1^
	Vitamin D_3_	810 (328)	723 (366)^2^	708 (423)^2^
	CaP+vitamin D_3_	657^a^ (318)	1149^b^ (426)^1^	1254^b^ (449)^1^
Plasma phosphate concentration [mmol/l]	CaP	1.1 (0.1)	1.2 (0.2)	1.1 (0.1)
	Vitamin D_3_	1.1 (0.1)	1.1 (0.2)	1.1 (0.1)
	CaP+vitamin D_3_	1.2 (0.1)	1.2 (0.1)	1.1 (0.2)
Renal phosphorus excretion [mg/d]	CaP	917 (354)	881 (289)	816 (289)
	Vitamin D_3_	857^a^ (236)	841^ab^ (276)	963^b ^(300)
	CaP+vitamin D_3_	807 (276)	818 (290)	894 (288)
Faecal phosporus excretion [mg/d]	CaP	554^a^ (382)	716^b^ (331)	754^b^ (331)^1^
	Vitamin D_3_	584 (218)	544 (309)	450 (275)^2^
	CaP+vitamin D_3_	496^a^ (229)	696^b^ (270)	740^b^ (260)^1^
Plasma magnesium concentration [mmol/l]	CaP	0.9 (0.1)	0.9 (0.1)	0.9 (0.1)
	Vitamin D_3_	1.0 (0.1)	0.9 (0.0)	0.9 (0.0)
	CaP+vitamin D_3_	0.9 (0.1)	0.9 (0.1)	0.9 (0.0)
Renal magnesium excretion [mg/d]	CaP	93 (41)	90 (45)	86 (31)
	Vitamin D_3_	79^a^ (23)	84^a^ (30)	98^b^ (31)
	CaP+vitamin D_3_	75 (31)	76 (25)	73 (26)
Faecal magnesium excretion [mg/d]	CaP	207 (136)	191 (103)	208 (119)
	Vitamin D_3_	234 (85)	242 (135)	205 (97)
	CaP+vitamin D_3_	191 (74)	178 (56)	180 (54)

n = 56; data are expressed as mean (standard deviation); CaP: tricalcium phosphate; ^a b^ mean values within a row with dissimilar superscript letters are significantly different (p ≤ 0.05); ^1 2^ mean values within a column with dissimilar superscript numbers are significantly different (p ≤ 0.05); results without superscripts in a row/column have no significant differences; effect of time was tested using general linear model with repeated measurements (with pairwise comparisons based on Fishers-LSD test); effect of supplementation was tested using univariate analysis of variance followed by Student-Newman-Keuls *post hoc* test.

There were no changes in plasma phosphate concentration in all intervention groups. After vitamin D_3_ supplementation, the renal phosphorus excretion significantly increased after eight weeks compared to placebo (p ≤ 0.05). Due to CaP supplementations (CaP and CaP + vitamin D_3_), the faecal excretion of phosphorus increased significantly after four and eight weeks compared to placebo. The faecal phosphorus excretion was significantly lower in the vitamin D_3_ group compared to the CaP and the CaP + vitamin D_3_ groups after eight weeks of intervention (p ≤ 0.05).

Both the plasma magnesium concentration and the faecal magnesium excretion did not change by any intervention. After vitamin D_3_ supplementation, the renal magnesium excretion significantly increased after eight weeks compared to four weeks and to placebo (p ≤ 0.05).

Plasma iron, ferritin, transferrin and transferrin saturation did not change due to any intervention (Table 4). After placebo and four weeks of intervention, the CaP + vitamin D_3_ group had significantly higher ferritin concentrations compared to the CaP group (p ≤ 0.05). The renal iron concentration was in most cases (67%) below the limit of detection (0.75 μmol/l) and was not subjected to statistical analysis.

**Table 4:** **Parameter of iron status after intervention with either tricalcium phosphate**, **vitamin D**_**3 **_**or both**

**Parameter**	**Supplements**	**Placebo**	**Interventions**	
			**4 weeks**	**8 weeks**
Plasma iron concentration [μmol/l]	CaP	18.8 (5.4)	18.3 (7.3)	19.5 (6.5)
	Vitamin D_3_	22.3 (5.2)	20.8 (6.8)	20.5 (5.1)
	CaP+vitamin D_3_	18.8 (6.7)	21.5 (5.2)	20.5 (5.5)
Plasma transferrin concentration [g/l]	CaP	2.5 (0.4)	2.4 (0.5)	2.5 (0.4)
	Vitamin D_3_	2.7 (0.5)	2.6 (0.5)	2.6 (0.6)
	CaP+vitamin D_3_	2.7 (0.4)	2.7 (0.4)	2.8 (0.6)
Transferrin saturation [%]	CaP	31.1 (10.1)	32.3 (16,8)	32.9 (10.9)
	Vitamin D_3_	35.0 (11.2)	24.1 (14.4)	33.6 (9.2)
	CaP+vitamin D_3_	29.1 (13.2)	32.0 (8.7)	31.3 (12.3)
Plasma ferritin [μg/l]	CaP	54.1 (28.4)^1^	47.5 (21.7)^1^	45.4 (22.6)
	Vitamin D_3_	88.3 (65.1)^12^	81.0 (60.1)^12^	72.6 (43.4)
	CaP+vitamin D_3_	134.6 (138.5)^2^	139.3 (151.3)^2^	105.0 (99.6)
Faecal iron excretion [mg/d]	CaP	8.7 (6.4)	7.7 (4.5)	7.7 (4.0)
	Vitamin D_3_	9.1 (4.0)	9.5 (5.0)	8.3 (3.7)
	CaP+vitamin D_3_	7.4 (2.9)	6.7 (2.4)	7.3 (2.9)

n = 51, except plasma transferrin n = 50; data are expressed as mean (standard deviation); CaP: tricalcium phosphate; ^1 2^ mean values within a column with dissimilar superscript numbers are significantly different (p ≤ 0.05); results without superscripts in a row/column have no significant differences; effect of time was tested using general linear model with repeated measurements (with pairwise comparisons based on Fishers-LSD test); effect of supplementation was tested using univariate analysis of variance followed by Student-Newman-Keuls *post hoc* test.

### Bone metabolism markers

**Figure 2:**
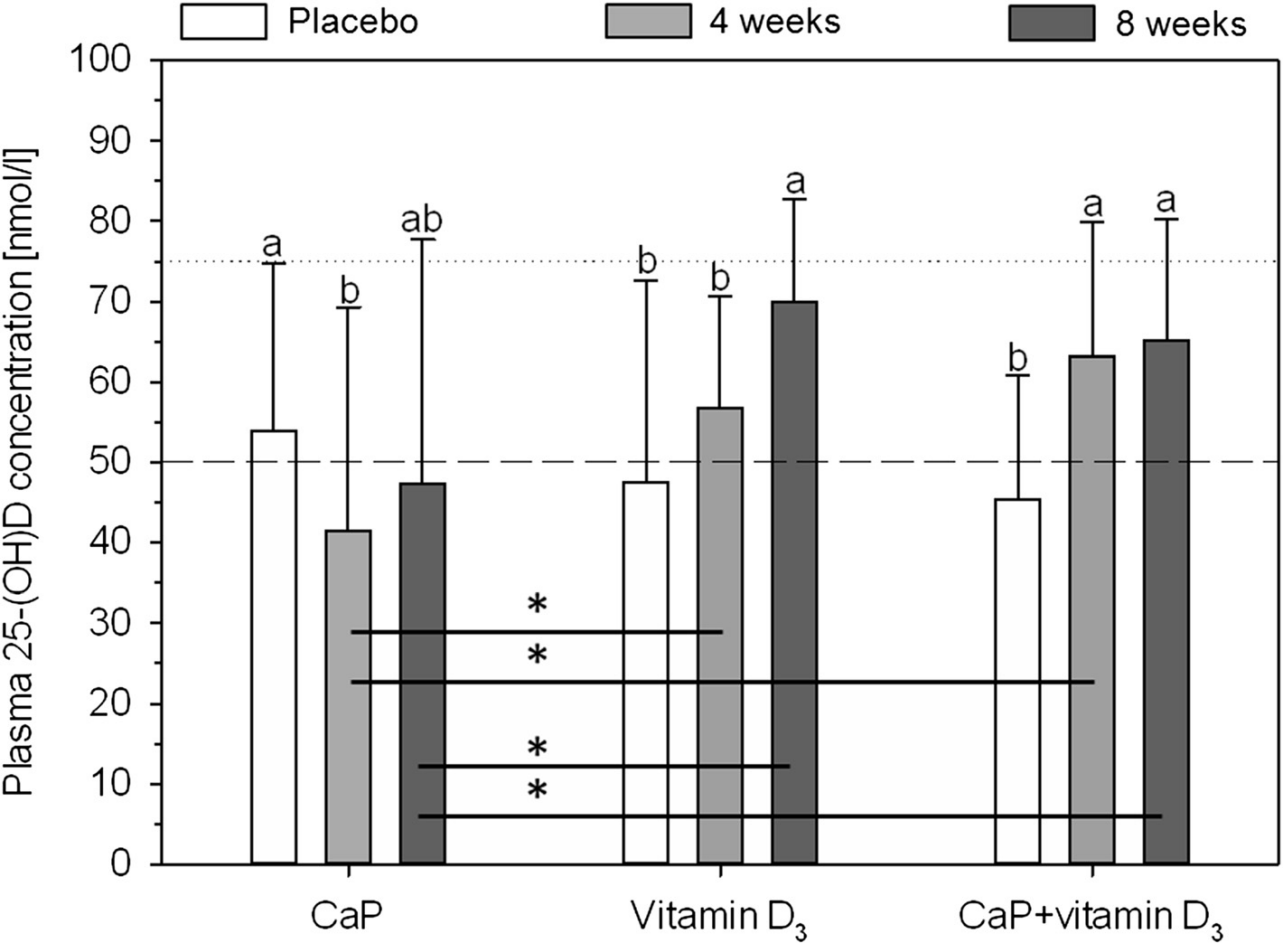
**Concentration of plasma 25-(OH)D after intervention with either tricalcium phosphate, vitamin D**_**3 **_**or both.** n =55; CaP: tricalcium phosphate; 25-(OH)D: 25-hydroxyvitamin D; broken line: recommended 25-(OH)D concentration; dotted line: optimal 25-(OH)D concentration; ^a b^ data are expressed as mean and standard deviation; mean values within a row with dissimilar superscript letters are significantly different (p ≤ 0.05); * significant difference between groups; effect of time was tested using general linear model with repeated measurements (with pairwise comparisons based on Fishers-LSD test); effect of supplementation was tested using univariate analysis of variance followed by Student-Newman-Keuls *post hoc* test.

Due to the vitamin D_3_ supplementation, the 25-(OH)D concentration in plasma significantly increased in the vitamin D_3_ group after eight weeks compared to placebo (Figure 2; p ≤ 0.05). The combined supplementation of CaP + vitamin D_3_ led to a significant increase after four and eight weeks of intervention compared to placebo (p ≤ 0.05). After CaP intervention, the plasma concentration of 25-(OH)D significantly decreased after four weeks compared to placebo (p ≤ 0.05). After four and eight weeks of intervention, the 25-(OH)D concentration was significantly higher in the vitamin D_3_ and CaP + vitamin D_3_ groups compared to the CaP group (p ≤ 0.05).

After four and eight weeks of CaP supplementation, 1,25-(OH)_2_D concentration in plasma decreased significantly compared to placebo (Table 5; p ≤ 0.05). After four weeks of CaP + vitamin D_3_ intervention, 1,25-(OH)_2_D concentration was significantly higher compared to placebo and eight weeks of CaP + vitamin D_3_ intervention (p ≤ 0.05). There were no changes in plasma PTH and calcitonin concentrations.

**Table 5:** **Bone metabolism markers and bone**-**related hormones in blood and urine after intervention with either tricalcium phosphate**, **vitamin D**_**3 **_**or both**

**Parameter**	**Supplements**	**Placebo**	**Interventions**	
			**4 weeks**	**8 weeks**
Plasma 1,25-(OH)_2_D [pmol/l]	CaP^t^	102.9^a^ (24.7)	93.3^b^ (24.7)	76.3^c^ (24.5)
	Vitamin D_3_^t^	97.9 (27.5)	108.3 (23.9)	99.2 (38.1)
	CaP+vitamin D_3_^t^	92.2^b^ (31.0)	100.4^a^ (31.1)	86.0^b ^(23.1)
Plasma osteocalcin [ng/ml]	CaP	20.3 (6.5)	20.4 (6.5)	20.9 (7.2)
	Vitamin D_3_	21.8 (7.2)	23.4 (8.2)	20.7 (8.8)
	CaP+vitamin D_3_	20.1 (7.0)	20.2 (6.4)	19.9 (7.6)
Plasma alkaline phosphatase	CaP	9.6^a^ (3.9)	10.7^b^ (3.9)	9.3^a^ (3.7)
	Vitamin D_3_	9.1^a^ (3.5)	10.3^b^ (3.6)	9.0^a^ (3.6)
	CaP+vitamin D_3_	9.8^a^ (3.4)	11.3^b^ (3.9)	9.5^a^ (3.1)
Serum PTH [μg/l]	CaP	56.6 (12.2)	74.4 (35.8)	71.9 (25.1)
	Vitamin D_3_	65.0 (24.7)	71.2 (29.2)	66.5 (21.0)
	CaP+vitamin D_3_	62.0 (20.1)	63.3 (17.8)	60.6 (19.8)
Serum CTX [ng/ml]	CaP	0.3 (0.1)	0.2 (0.1)	0.3 (0.2)
	Vitamin D_3_	0.3 (0.2)	0.3 (0.2)	0.3 (0.1)
	CaP+vitamin D_3_	0.3 (0.1)	0.2 (0.1)	0.2 (0.1)
Serum P1NP [ng/ml]	CaP	44.5 (19.1)	42.5 (16.5)	44.2 (19.7)
	Vitamin D_3_	48.8 (18.9)	44.4 (20.7)	50.9 (24.7)
	CaP+vitamin D_3_	41.4 (17.6)	36.3 (19.7)	40.4 (15.9)
Urine DPD [nmol/l]	CaP	29.2 (14.9)	31.6 (20.0)	27.0 (14.5)
	Vitamin D_3_	25.3 (13.7)	22.8 (11.5)	25.5 (9.8)
	CaP+vitamin D_3_	23.7 (9.0)	25.8 (16.0)	28.1 (15.2)
Urine CTX [mg/l]	CaP	1.0 (0.6)	1.1 (1.1)	0.7 (0.5)
	Vitamin D_3_	0.9 (0.7)	0.8 (0.6)	1.1 (0.6)
	CaP+vitamin D_3_	0.9 (0.7)	0.9 (0.7)	1.2 (1.1)

n = 55; data are expressed as mean (standard deviation); CaP: tricalcium phosphate; 1,25-(OH)_2_D: 1alpha,25-dihydroxyvitamin D; 25-(OH)D: 25-hydroxyvitamin D; *PTH*: parathormone; CTX: cross-linked C-terminal telopeptide of type I collagen; P1NP: N-terminal propeptide of type I procollagen; *DPD*: desoxypyridinoline; ^a b^ mean values within a row with dissimilar superscript letters are significantly different (p ≤ 0.05); results without superscripts in a row/column have no significant differences; effect of time was tested using general linear model with repeated measurements (with pairwise comparisons based on Fishers-LSD test); effect of supplementation was tested using univariate analysis of variance followed by Student-Newman-Keuls *post hoc* test; ^t^ for statistical analysis the data were log_10_ transformed, because of variance heterogeneity.

Bone resorption markers, serum and urinary CTX and urinary DPD did not change due to the three interventions (Table 5). The bone formation markers plasma osteocalcin and serum P1NP did not change after any intervention. All supplementations led to a significant increase of plasma alkaline phosphatase concentration after four weeks compared to placebo and to eight weeks (Table 5, p ≤ 0.05).

## Discussion

The additional intake of 1 g calcium (via CaP) per day was used in other studies due to beneficial effects on intestinal metabolism [10,13]. The additional daily intake of 10 μg vitamin D_3_ was chosen, because 5 μg/d was the previously recommended vitamin D intake in Germany [14]. In 2012, the German Nutrition Society set up this value to 20 μg/d for adolescents and adults under 65 years [15], because the UV index in Germany is lower than 3 for about six months of the year [16]. A rule of thumb says, that indexes lower than 3 are insufficient to produce vitamin D in the skin [16]. In the present study, the total intake of vitamin D (supplement, defined diet and bread) was approximately 15 μg/d in the vitamin D_3_-supplemented groups, and therefore below the actual recommendation of the German Nutrition Society [15].

However, after vitamin D_3_ supplementation, the 25-(OH)D concentration significantly increased after eight weeks (Figure 2). The mean increases in the vitamin D_3_ and in the CaP + vitamin D_3_ groups were 22.5 nmol/l and 19.8 nmol/l, respectively (Figure 2). The present results indicate, that the supplementation with 10 μg/d was enough to increase 25-(OH)D significantly. Only 5% of participants treated with vitamin D_3_ had 25-(OH)D concentrations below 50 nmol/l after eight weeks of intervention. Furthermore, the mean increase is in accordance with other vitamin D_3_ supplementation studies [17,18].

Interestingly, in the CaP + vitamin D_3_ group the increase in 25-(OH)D was significant after four and eight weeks, but in the vitamin D_3_ group only after eight weeks (Figure 2). It seems that the combination with CaP leads to a faster increase in 25-(OH)D concentration. A low calcium intake could lead to a higher turnover of vitamin D metabolites (higher production of 1,25-(OH)_2_D, due to PTH increase), while a high calcium intake could be 25-(OH)D sparing [19]. These findings were based on rat experiments, in which the half-life of 25-(OH)D was longer when calcium intake was high. Furthermore, in a rat experiment conducted by Anderson *et al*., animals fed high calcium combined with vitamin D_3_ had significantly higher serum 25-(OH)D concentrations compared to animals fed low calcium combined with vitamin D_3_[20]. In the present study, plasma calcium and PTH were not significantly affected by the interventions. Only the 1,25-(OH)_2_D concentration decreased compared to placebo after eight weeks of CaP (CaP alone and CaP + vitamin D_3_) supplementations. From the increase of 25-(OH)D concentration after four weeks of CaP + vitamin D_3_ supplementation it can be concluded, that the combination of vitamin D_3_ and calcium is more effective than vitamin D_3_ alone.

In the present study, we determined different biomarkers of bone metabolism, like urinary CTX, serum CTX and urinary DPD for bone resorption and plasma alkaline phosphatase, serum P1NP and plasma osteocalcin for bone formation. None of the bone metabolism markers changed related to the CaP, vitamin D_3_ or CaP + vitamin D_3_ supplementations. Literature indicates that a modulation of bone metabolism through calcium and/or vitamin D supplementations occurred especially in older subjects with a vitamin D deficiency [12,21]. In studies involving subjects with no or only marginal vitamin D deficiency, no beneficial effects on bone metabolism were observed [22,23].

Due to CaP supplementation, the faecal excretion of calcium and phosphorus significantly increased. This is based on the formation of amorphous calcium phosphate, a well-known process in the intestine [8,24]. Thus, only a minor part of the calcium can be absorbed [11]. The unchanged excretion of calcium and phosphorus in urine and the comparable plasma concentration confirm this suggestion. Vitamin D is known to increase the absorption of calcium and phosphate in the human gut, in order to maintain the calcium homeostasis. After supplementation of vitamin D_3_ alone, only the renal excretion of phosphorus significantly increased after eight weeks compared to placebo. In phosphorus equilibrium, the amounts of intestinally absorbed phosphorus is equal to the excreted phosphorus by the kidney [25]. Thus, the increased excretion indicates higher phosphorus absorption due to vitamin D_3_ supplementation.

In a rat experiment, it has been shown that magnesium is also able to form an insoluble complex with calcium and phosphate [26]. When this formation occurs in the human gut, the absorption of magnesium would be inhibited. After supplementation with high amounts of phosphorus, Greger *et al*. showed a significant increase in faecal excretion of magnesium, but simultaneously a decrease of magnesium in urinary excretion [27]. The authors concluded that magnesium metabolism was not negatively influenced by high phosphorus doses [27]. In the present study, CaP supplementation had no harmful effects on magnesium metabolism. After eight weeks of vitamin D_3_ supplementation, the renal excretion of magnesium increased compared to placebo. Hardwick *et al*. reviewed the literature concerning magnesium absorption and suggested that a significant amount of magnesium absorption is vitamin D-dependent [28].

Literature indicates, that calcium supplementation can decrease iron absorption, both from haem and non-haem iron [29]. This decrease in iron absorption has been shown mostly in short-term studies [30-32]. In contrast, Minihane and Fairweather-Tait supplemented calcium carbonate in a short-term (1200 mg calcium carbonate for one day) and in a long-term study (1200 mg calcium carbonate over six months) and showed that the non-haem iron absorption decreased only in the short-term supplementation [33]. By supplementing 1200 mg calcium as calcium carbonate over six months, they found no changes in any of the haematologic indexes (i.e. haemoglobin, plasma ferritin). The mechanism proposed by the authors is an adaptive response in the intestinal mucosa cell: the lower supply of iron to plasma after calcium intake may modify the developing enterocytes in order to stimulate specific proteins [33]. Thus, the dietary iron is used more efficiently, when developing cells are mature [33]. The unchanged results of faecal iron and of iron status on the present study indexes (transferrin, transferrin saturation, ferritin) confirm the results of Minihane and Fairweather-Tait, that long-term CaP supplementation does not negatively influence the parameters of iron metabolism. The significantly higher ferritin concentration in the CaP + vitamin D_3_ is due to two participants with concentrations in the upper limits of normal. In all, the presented results underline that supplementation with CaP and/or vitamin D_3_ did not negatively influence the metabolism of magnesium and iron.

## Conclusion

The results of the present human study shows that supplementation with 10 μg vitamin D_3_ significantly increases 25-(OH)D. Furthermore, a combination with 1 g calcium (as CaP) beneficially influences the increasing effect on the 25-(OH)D concentration. Both CaP alone or in combination with vitamin D_3_ has no beneficial or harmful effects on bone metabolism markers and on the metabolism of calcium, phosphorus, magnesium and iron in middle-aged healthy persons.

## Abbreviations

CaP: Tricalcium phosphate; CTX: Cross-linked C-terminal telopeptide of type I collagen; DPD: Desoxypyridinoline; PTH: Parathormone; P1NP: N-terminal propeptide of type I procollagen; 1,25-(OH)2D: 1alpha,25-dihydroxyvitamin D; 25-(OH)D: 25-hydroxyvitamin D.

## Competing interests

The authors declare that they have no competing interests.

## Authors’ contributions

UT conducted research, wrote the manuscript and performed statistical analysis; UT, NN, ML and JK were involved in analyses; UT and GJ designed research and had primary responsibility for final content. All authors read and approved the final manuscript.
